# Programmable microfluidics for dynamic multiband camouflage

**DOI:** 10.1038/s41378-023-00494-3

**Published:** 2023-04-04

**Authors:** Chunzao Feng, Mingran Mao, Xiaohui Zhang, Yutian Liao, Xiaohui Xiao, Huidong Liu, Kang Liu

**Affiliations:** MOE Key Laboratory of Hydraulic Machinery Transients, School of Power and Mechanical Engineering, Wuhan University, Wuhan, 430072 Hubei China

**Keywords:** Optical materials and structures, Nanoscience and technology

## Abstract

Achieving multiband camouflage covering both visible and infrared regions is challenging due to the broad bandwidth and differentiated regulation demand in diverse regions. In this work, we propose a programmable microfluidic strategy that uses dye molecules in layered fluids to manipulate visible light- and infrared-semitransparent solvent to manipulate infrared light. With three primary fluid inputs, we achieve 64 chromaticity values and 8 emissivities from 0.42 to 0.90. In view of the wide tuning range, we demonstrate that the microfluidic film can dynamically change its surface reflectance to blend into varying backgrounds in both visible and infrared images. Moreover, we fabricate the microfluidic device in a textile form and demonstrate its ability to match exactly with the colors of natural leaves of different seasons in the full hyperspectrum range. Considering the broadband modulation and ease of operation, the programmable microfluidic strategy provides a feasible approach for smart optical surfaces in long-span optical spectra.

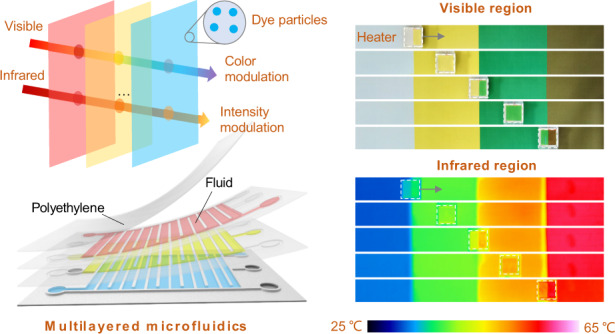

## Introduction

The ability of certain animals to change physical appearance plays multifunctional roles in predation, self-protection and other physiological behaviors^1–4^. Such ability has fascinated humans for centuries and inspired the development of camouflage materials and devices^5^. Plasmonic nanodot arrays^6^, responsive photonic crystals^7,8^ and multilayered thermochromic liquid crystals^9^ have been designed to selectively reflect light in the visible spectrum to change their colors and blend into backgrounds. Electrochromic devices^10–12^, thermochromic materials^13,14^ and mechanochromatic systems^15^ can dynamically alter their infrared emissivity in response to ambient temperature variation. For all these optical manipulation technologies, a general challenge is the tuning range^16–18^. In terms of visible light, sufficiently tunable color without chromatic aberration is difficult to achieve. A large infrared tuning range is also challenging to achieve due to the limitations of materials to respond to external fields. Moreover, multiband camouflage covering both the visible and infrared light spectra is even more difficult to achieve since visual color tuning relies on frequency manipulation, while infrared tuning relies on intensity changes.

Optofluidic technologies use light-fluid interactions to achieve fluid flow or optical manipulation for different applications, such as light tuning^19^ and sensing^20,21^. In 2012, Morin et al. proposed the idea of using microfluidic networks for camouflage and display in soft machines^22^. As a camouflage and display technology, microfluidics has two apparent advantages compared to existing electrochromic, mechanochromatic and thermochromic approaches. First, sufficient color switching can be achieved due to the large optical index contrast induced by fluid replacement^22–25^. However, a wide color range requires complex accessory reservoirs and pumping systems^26^. Second, the fluid contains solutes or particles along with solvents^27–29^, which provides possible dual-functional modulation for the demand in different spectrum regions. Nevertheless, this superior characteristic has not been explored previously. Therefore, to apply microfluidics in practical and powerful camouflage and display, simple structural design, easy color mixing strategy and further optical exploration of different substances in fluids are still crucial.

Herein, we propose a microfluidic strategy that employs a multilayered fluidic structure and dual-functional fluids to implement dynamic camouflage in both the visible and infrared regions. Within the fluids, dye molecules selectively absorb visible light, and the solvent is semitransparent to infrared light. With a three-layered structure, three primary fluids of red, yellow and blue can be controlled programmatically to achieve almost full-visible colors and different infrared reflective intensities, and to resemble varying background colors and temperatures. In addition, we fabricated the microfluidic device in a textile form and demonstrated its ability to match leaves of different seasons in full hyperspectrum range, showing the application potential of programmable microfluidics in adaptive camouflage, broadband display and active thermal management.

## Results and discussion

**Fig. 1:**
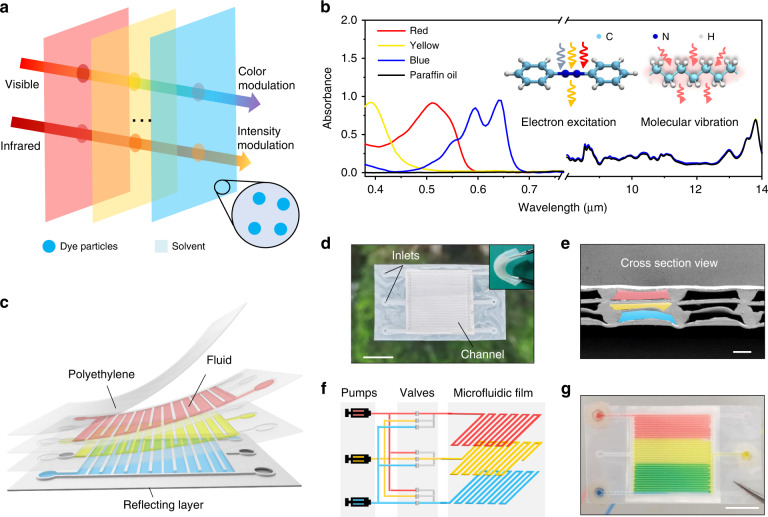
Light modulation principle and structure of the microfluidic film. **a** Schematic of the multilayered microfluidics. **b** Spectral absorbance in the visible and mid-infrared regions of paraffin oil and fluids with red (C_23_H_16_ClN_3_O_2_), yellow (C_16_H_9_N_4_O_9_S_2_Na_3_) and blue (C_32_H_18_N_8_) dyes. The thicknesses of all the fluids were ~100 μm. The left inset shows the selective absorption of visible light by electron excitation in azobenzene molecules. The right inset shows weak infrared absorption via vibration in alkane molecules. **c** Schematic showing a three-layered microchannel with a back reflecting layer. **d** Photograph of the original film (~2.5 cm × 2.5 cm) without solution inside. Scale bar, 1 cm. The inset shows the film at a bending angle of ~50°. **e** Cross-sectional SEM images of the film. Scale bar, 100 μm. **f** Programmable manipulation of the fluids via the pumps and valves. **g** Photograph of the film partially filled with fluids in the three layers. Scale bar, 1 cm

As shown in Fig. 1a, the programmable microfluidic design consists of multilayered fluids with dye molecules and infrared-semitransparent solvent. In each layer of fluid, visible light is selectively absorbed through electron excitation in dye molecules, and infrared light is partly absorbed via molecular vibration in the solvent (Fig. 1b)^30^. Through the separate mechanism, the microfluidic design provides an approach to manipulate visible and infrared light without mutual interference between them. With the multilayered structure, a broad range of colors was achieved through the combination of several kinds of initial colors. Infrared absorbance can be controlled by the effective thickness of the solvent. The emissivity can thus be modulated when combined with a low-emissive substrate. Theoretically, the Infrared emissivity tuning range can be as wide as 0–1.

To demonstrate this idea, we fabricated a device with three-layered microchannels, as shown in Fig. 1c. The microchannel ensured quick and complete fluid filling and removal. Polyethylene (PE) was used to construct the channels due to its transparency to light from the visible to the end of the mid-infrared light spectrum, except for the infrared absorptions in the fingerprint region (Fig. S1). Paraffin oil was employed as the solvent due to its visible transparency and infrared semitransparency. Paratonere (C_23_H_16_ClN_3_O_2_), tartrazine (C_16_H_9_N_4_O_9_S_2_Na_3_), and phthalocyanine (C_32_H_18_N_8_) were added to the paraffin oil to obtain three primary colors of red, yellow and blue, respectively (Fig. S2). The content of the dyes was low enough to avoid interference with the mid-infrared absorption of the oil (“Methods” and Fig. 1b). We also deposited silver films on both sides of the bottom layer to provide high reflectivity (Fig. S3). The PE microchannels were fabricated by a stainless-steel mold (Fig. S4) and bonded by hot pressing. Details about the fabrication can be found in the “Methods” and Fig. S5.

Figure 1d shows a photograph of the as-fabricated thin film, which is flexible (Fig. 1d inset) with a white appearance due to the reflection of silver and scattering of the channels. Here, it is important to note that the heights of the channels were ~80, 40 and 100 μm from the top layer to the bottom layer (Figs. 1e and S6). The height difference provides an additional variable for visible and infrared regulation. For further intelligent control, the fluidic flow in each layer can be programmatically controlled by pumps and valves, as schematically shown in Fig. 1f and Supplementary Video 1. Figure 1g shows a photograph of the thin film partially filled with fluids of three colors, showing its good capacity for coloration and color mixing.

**Fig. 2:**
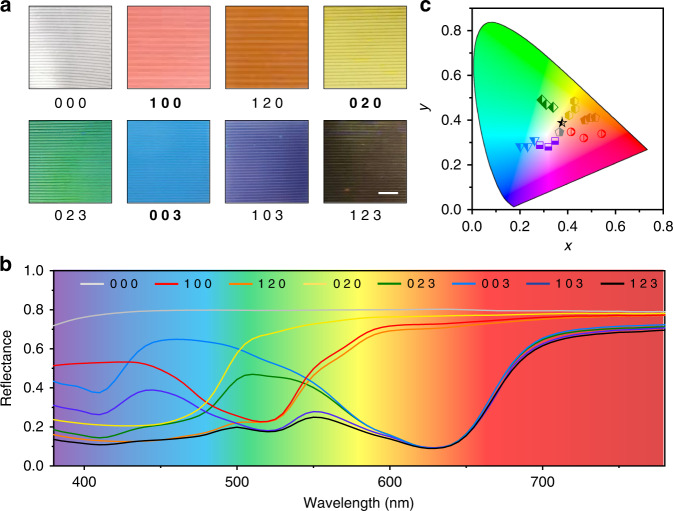
Visible colors of the microfluidic film at different filling states. **a** Typical photographs of the film in states represented by different arrays. **b** Measured spectral reflectance in the visible region corresponding to the photographs in **a**. **c** Chromatic diagram of the displayed colors

We investigated the color regulation ability of the thin film device with three primary colors: red, yellow and blue (Fig. 2). For simplicity and ease of coding, an array of (a, b, c) was used to present the states of fluids in the top, middle and bottom layers of the device. Each letter had an optional value of 0, 1, 2 or 3. Here, 0 represented no fluid, and 1, 2 and 3 represented red, yellow and blue fluids, respectively. As shown in Fig. 2a, the states of (1 0 0), (0 2 0) and (0 0 3) represented the original red, yellow and blue colors. The lightness of the primary colors can be adjusted by stacking the same fluid, for example, (1 1 0), (2 0 2) and (3 3 3) (Figs. 2c and S7). With the state of (0 0 0), (1 2 0), (0 2 3), (1 0 3) and (1 2 3), white, orange, green, violet and brown were also obtained. Here, the color mixing followed the subtractive color mixing law (Fig. S8)^31^. The thickness of the microchannel in each layer was less than 100 μm to ensure transparency at each wavelength within the visible region (Fig. S9). Different from traditional color mixing with a dye mixture^32^, this approach used light subtraction from each layer without dye mixing and thus allowed easier reversible color manipulation (Supporting Video 1). The measured reflectance spectra of the film at different states are shown in Fig. 2b. More states with other colors are listed in Table S1. Based on these spectra, we calculated the chromaticity of each color (Supplementary Note 1). The results cover a broad range of colors, as shown in Fig. 2c, and can be further broadened by adding microchannel layers.

**Fig. 3:**
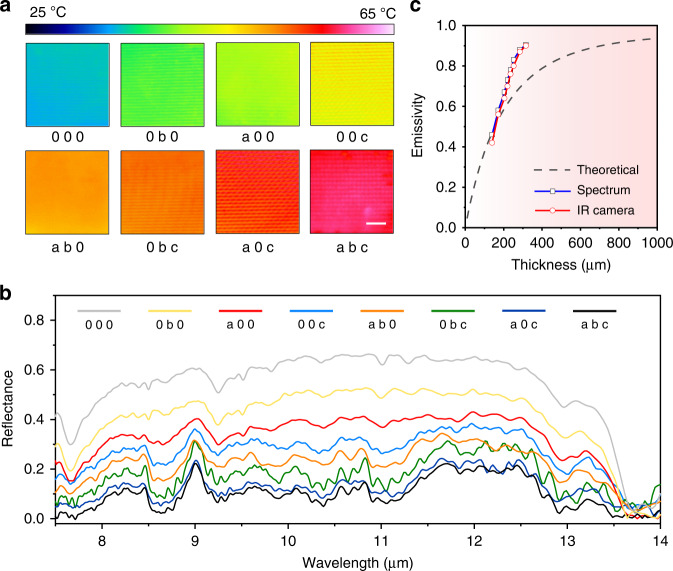
Infrared manipulation of the microfluidic film. **a** Thermal images of the film at different states with an applied temperature of 60 °C. **b** Spectral reflectance in the mid-infrared range corresponding to the states in **a**. **c** Measured thermal emissivities of the film with different thicknesses of the fluid. The calculated thermal emissivities of PE films with different thicknesses are plotted for comparison

Figure 3 shows the infrared manipulation of the microfluidic device. As shown in Fig. 3a, the infrared images of the film are apparently different with different filling states under the same temperature of 60 °C (Fig. S10). By adjusting the emissivity of the infrared camera until the film displaying the real temperature (60 °C), we obtained the emissivity of the film (Fig. S10 and “Methods”). As shown in Fig. 3c, the emissivity increases from 0.42 to 0.90 with different filling states. To confirm the results, we measured the reflectance spectra of the film. As shown in Fig. 3b, the reflection spectra show almost the same shape but different intensities. By integrating the spectral absorption radiation over the wavelength with respect to black body radiation, we obtained the integrated emissivity of the film in the atmosphere transparent window of 7.5–14 μm. The results coincide well with the values from thermographic camera measurements (Fig. 3c).

To further illustrate the infrared performance of the device, we calculated the emissivity of PE films with different thicknesses. Details about the calculation can be found in Supplementary Note 2 and Fig. S11. As shown in Fig. 3c, the emissivity value of the device without fluid is close to the theoretical value of the PE film. With the filling of dye fluid, the emissivity of the device becomes higher than that of a PE film with the same thickness because of the higher absorption of paraffin oil than that of solid PE^30^. The emissivity tuning range of ~0.48 is comparable to the best values of other approaches^11,12,15^. However, this value is not the upper limit of this film. According to Fig. 3c, the tuning range is restricted by the initial emissivity of the film (0.42) which corresponds to an equivalent thickness of ~140 μm (Fig. S6). Hence, further reducing the thickness of the supporting layer or increasing the inherent infrared transparency of the channels would enlarge the tuning range.

It is also noted that the three primary fluids have very similar infrared absorbances (Fig. 1b) over the spectrum range of 8–14 μm. The infrared emissivities of different color fluids with the same thickness are thus similar. Hence, for a certain infrared emissivity, we have various color choices, as listed in Table S1. For the same color, the infrared emissivity could also switch between different values by filling the same fluid into different layers of the channels. Since the thickness of the microchannels are different, for red, yellow and blue colors, we had seven values to choose from. Different from previously reported methods regarding multiband spectra^12,33^, the visible and infrared manipulation here can be simultaneously and separately controlled.

**Fig. 4:**
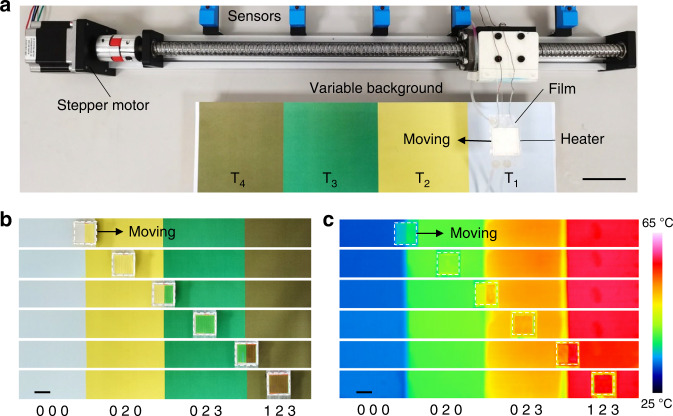
Dynamic visible and infrared camouflage of the microfluidic film. **a** Experimental setups to demonstrate camouflage performance. Scale bar, 5 cm. Visible (**b**) and infrared (**c**) images showing the film changing its surface color and emissivity across backgrounds with different colors and temperatures. Scale bar, 2 cm

In view of the tunable visible and infrared appearance, we demonstrated the application potential of our microfluidic thin film in visible and infrared camouflage. As shown in Fig. 4a, we attached a microfluidic film with a size of 2.5 cm × 2.5 cm on a white ceramic heater with a temperature of 60 °C. The heater moved across an artificial background with the color varying from white to yellow, green, and brown. The temperatures were 30, 38, 45, and 55 °C. A stepper motor controlled the moving speed of the heater. Five position sensors located the heater and controlled the programmable pumps and valves to drive the fluid flow in the film. A digital camera and an infrared camera were used to capture the visible and infrared images of the heater. As shown in Fig. 4b, c, the heater adaptively changed its color to match the varying background in both visible and infrared imaging (Supplementary Videos 2 and 3), achieving dynamic visible and mid-infrared camouflage. For the heater without the microfluidic film, both cameras clearly captured the moving heater (Fig. S12a, b).

**Fig. 5:**
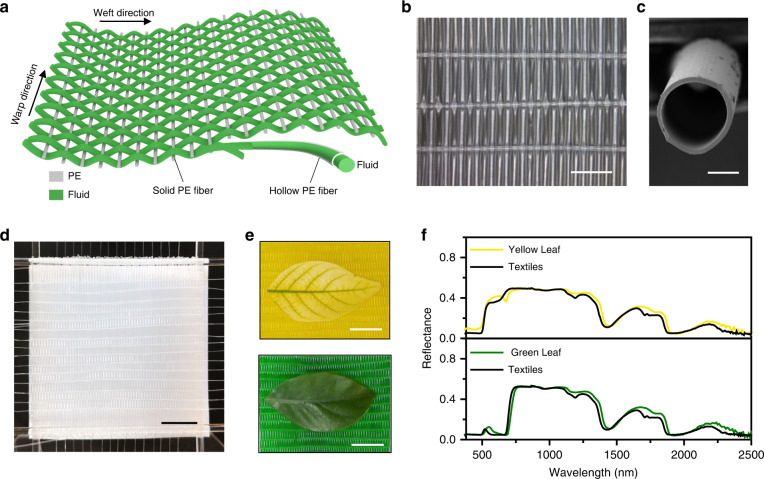
Hyperspectral camouflage performance of the microfluidic textiles at 400–2500 nm. **a** Schematic showing the structure of the microfluidic textiles constructed by hollow PE fibers and solid PE fibers. **b** Photograph of the woven textiles showing the weft yarn and warp yarn. Scale bar, 2 mm. **c** Cross-sectional SEM image of a hollow PE fiber. Scale bar, 200 μm. **d** Photograph of an original two-layered textile without fluids inside (5 × 5 cm^2^). Scale bar, 1 cm. **e** Photographs of the microfluidic textiles with yellow and green leaves. Scale bar, 1 cm. **f** Reflectance spectra of the textiles and natural leaves in the hyperspectrum range of 400–2500 nm

To show the application potential of the microfluidic film, we further fabricated the film in a textile form (Fig. 5a). Commercial PE hollow fibers were used as the weft yarn, and solid PE fibers were used as warp yarns to weave the textile (Fig. 5a). The hollow fiber bent at a small angle across the solid fiber (Fig. 5b). The inner diameter of the hollow fiber is 300 μm, and the thickness of the wall is 25 μm (Fig. 5c). The diameter of the solid fiber is ~250 μm (Fig. S13). Figure 5d shows a photograph of the fabricated textile with a size of 5 × 5 cm^2^. Further scaling up can be realized through industrial technique optimization.

Microfluidic textiles were demonstrated for ground hyperspectral camouflage in the solar radiation spectrum of 400–2500 nm^34^. As a common ground vegetation with complex optical characteristics^35^, natural leaves were applied as the background (Supplementary Note 3). We designed two-layered textiles to simulate the reflectance spectrum of the leaves (Fig. S14). The top layer with colored water was used for visible color display, and the bottom layer with gray water was used for reflectance intensity modulation. White paper was placed at the bottom to enhance the reflection intensity. As presented in Fig. 5e, f, the textile filled with designed fluids not only demonstrates very similar color to the green leaves, but also presents a similar reflection spectrum. By changing the fluid inside the textiles, the color and spectrum immediately changed to be close to that of yellow leaves, suggesting the adaptability of the camouflage film in response to season or location variation in the natural environment^36^. The two-layered microchannel film shows camouflage performance similar to that of the textiles (Figs. S15 and S16). However, the pumping rate of the microchannel was 5 cm s^−1^ bar^−1^, much smaller than the 9 cm s^−1^ bar^−1^ for the textile (Fig. S17). Hence, the textile may allow for quicker switching than rectangular microchannels with the same flow rate.

In summary, we have developed a programmable microfluidic device with a multilayered fluidic structure and dual-functional fluids, which can dynamically display nearly the entire visible color and infrared emissivity in the range from 0.42 to 0.90 with three primary fluid inputs. This capacity endows the device with application potential in both visible and mid-infrared camouflage. Moreover, we fabricated the microfluidic film in a textile form for scale-up applications. With a specifically designed fluid and stack structure, the textile dynamically matches reflectance of leaves in the full spectrum from the visible to near- infrared region. Considering broadband and delicate light modulation, programmable microfluidics may open up a new way for smart optical surfaces across a multiband electromagnetic spectrum and have great potential in applications such as adaptive camouflage, broadband display and active thermal management.

## Materials and methods

### Preparation of fluids

Red, yellow, and blue oil-based fluids were prepared by mixing the commercial dyes paratonere (C_23_H_16_ClN_3_O_2_), tartrazine (C_16_H_9_N_4_O_9_S_2_Na_3_), and phthalocyanine (C_32_H_18_N_8_) with low-viscosity paraffin oil (YNSOL-IP60, Yitai Co.) (Fig. S2). The mass ratios of dye and paraffin oil in the three fluids were 1:100. The yellow and green aqueous fluids were prepared by mixing the commercial yellow and green inks (8501 and 8902S, Semiramis) with deionized water at a volume ratio of 1:1. Two gray aqueous fluids were prepared by mixing commercial black ink (8101, Semiramis) with deionized water at different volume ratios of 1:10 and 1:20 to match the water content of natural green and yellow leaves.

### Fabrication of the microfluidic films

Fabrication of the three-layered film involved three subprocesses (Fig. S5): thermocompression molding, Ag deposition, and thermocompression bonding. Each layer of the microchannel layers was fabricated by thermocompression molding. The mold was fabricated by chemical etching on a stainless-steel sheet (Fig. S4a). The width and depth of the channels were 200 and 130 μm, respectively (Fig. S4b, c). High-density polyethylene (HDPE) particles (density of 0.95 g cm^−3^, melting point of ~130 °C) were placed on a stainless-steel mold and hot-pressed for 10 min to form the microchannel. The heating temperature was 150 °C, and the applied pressure was 10 MPa. A low-density polyethylene (LDPE) (density of 0.92 g cm^−3^, melting point of ~120 °C) cover layer was also fabricated by hot-pressing with two plane stainless-steel sheets. Spacers were placed around the stainless-steel mold to control the thickness of each layer. Inlet or outlet holes were punched at designated positions in each layer. Ag layers were deposited on both sides of the single-side channel layer (Fig. S5b) by magnetic sputtering (JSD350-II, Jiashuo). To avoid the effect of Ag deposition on the bonding of different layers, a protection layer was deposited on the surface of the channels by pressing the microchannel layer on carbon transfer paper. After Ag deposition, the protection layer was washed away by alcohol (EtOH). The prepared layers were bonded as schematically shown in Fig. S5c. The two-sided channel layer was covered by two LDPE layers on each side and hot-pressed at 120 °C for 30 min under a pressure of 0.8 MPa. The obtained film was then bonded with the aforementioned Ag-sputtered layer at 120 °C for 30 min under a pressure of 0.6 MPa, obtaining the three-layered film.

### Integrated thermal emissivity measurement

Figure S10a shows the setup for measuring the integrated thermal emissivity. The film was heated to a temperature of 60 °C (Fig. S10b). A sheet of PVC tape with an emissivity of 0.91 was stuck on the surface of the film as a reference. A thermal camera was used to capture the temperature distribution, as shown in Fig. S10b. The real temperature was obtained from the PVC tape by setting the emissivity as 0.91. By changing the emissivity in the infrared camera until the microfluidic film exhibited the real temperature, the integrated emissivity of the film was obtained. During the measurements, the room temperature was maintained at ~18 °C, and the relative humidity was maintained at ~60%.

### Dual-band camouflage performance demonstration

The microfluidic film was placed on a ceramic heater. The heater was connected to a mobile station at the guide rail via a piece of acrylic plate. The movement of the station was controlled by a stepping motor (57HSZ1.2N). Four papers with different colors (10 cm × 10 cm) were stuck on four pieces of Al plates, which were placed on four silicon heaters. Five sensors were fixed at different positions along the guide rail, which were placed at five boundaries of the Al plates. Three peristaltic pumps (Ditron-tech BT100-3W) controlled the fluidic flow in the microchannels. The whole system was controlled by a computer through the RS 485 communication protocols. When the camouflage heater moved across the background, five sensors located the heater and controlled the pumps to change the optical appearance of the film. The fluids were preset according to the arrays in Fig. 4b. The flow rate of the fluid matched the moving speed of the heater across the boundaries between different background colors. A digital camera (DMC-GH4, Lumix) and an infrared camera (TiX640, Fluke) were used to simultaneously record the camouflage performance.

### Characterizations

The cross-sectional structures of the empty films and fibers were characterized by a scanning electron microscope (TESCAN, MIRA3). The spectral reflectances of the films, textiles and leaves in the wavelength range of 300–2500 nm were measured by a UV‒Vis-NIR spectrophotometer (Lambda 1050, Perkin Elmer) equipped with an integrating sphere (Labspher8). A glass cuvette with an optical path of 10 mm was used to measure the transmittance of the fluids. Infrared spectra of the film were measured by a Fourier transform infrared spectrometer (FTIR, INVENIO S, Bruker) with a gold integrating sphere (A562). An infrared transparent cell consisting of two infrared transparent KBr cell windows separated by a ring-shaped spacer was applied to measure the infrared transmittance of the fluids (Fig. S18). Infrared images were taken by a thermal infrared camera working in the wavelength range of 7.5–14 μm (Fluke TiX 640). The sizes of the microchannels were characterized by a 3D optical surface profiler (ZYGO NewView™ 9000).

## Supplementary information

Supplementary Information

Supplementary Video 1

Supplementary Video 2

Supplementary Video 3

## Data Availability

The data that support the findings of this study are available from the corresponding author upon reasonable request.
