# Supplementary material for: Programmable microfluidics for dynamic multiband camouflage
Source: Microsyst Nanoeng. 2023 Apr 4;9:43. doi: 10.1038/s41378-023-00494-3 (PMC10073183; doi:10.1038/s41378-023-00494-3)
Supplement: Supplementary file 1 — Supplementary Information [file 41378_2023_494_MOESM1_ESM.docx]

Supplementary Information

Programmable Microfluidics for Dynamic Multiband Camouflage

Chunzao Feng, Mingran Mao, Yutian Liao, Xiaohui Zhang, Xiaohui Xiao, Huidong Liu^*^, Kang Liu*

^1^MOE Key Laboratory of Hydraulic Machinery Transients, School of Power and Mechanical Engineering, Wuhan University, Wuhan, Hubei 430072, China

*Correspondence to: [kang.liu@whu.edu.cn](mailto:kang.liu@whu.edu.cn), liuhuidong30@whu.edu.cn

**Including:**

Supplementary Notes 1 to 3.

Supplementary Figures 1 to 18.

Supplementary Table 1.

Supplementary Video 1-3.

**Supplementary notes**

**1. Chromaticity calculations**

The color perceived by human eyes depends on the sensitivity of the eye’s three cone cells, the spectral reflection (*R_λ_*) from the microfluidic film, and the spectral intensity of the light source (*I_λ_*). The sensitivity of the eye’s three cone cells can be represented by three color matching functions, *x_λ_*, *y_λ_* and *z_λ_*, which were established from experiments on a standard observer by the International Commission On Illumination (CIE) in 1931^1^. The CIE tristimulus values *X*, *Y*, and *Z* can be calculated as:

$X=100\frac{\int I_{\lambda}R_{\lambda}x_{\lambda}d\lambda}{\int I_{\lambda}x_{\lambda}d\lambda}$ (1)

$Y=100\frac{\int I_{\lambda}R_{\lambda}y_{\lambda}d\lambda}{\int I_{\lambda}y_{\lambda}d\lambda}$ (2)

$Z=100\frac{\int I_{\lambda}R_{\lambda}z_{\lambda}d\lambda}{\int I_{\lambda}z_{\lambda}d\lambda}$ (3)

$I_{\lambda}$ is taken as the solar spectral intensity. The chromaticity of the color of the microfluidic film is determined by two normalized parameters:

$x= \frac{X}{X+Y+Z}$ (4)

$y= \frac{Y}{X+Y+Z}$ (5)

Based on the spectral reflection of the microfluidic film, we can calculate the values of the two parameters *x* and *y*. Then we can find the corresponding chromaticity in CIE 1931 color space, as shown in Fig. 2c in the main text.

**2. Theoretical calculations of the emissivity of PE**

We calculated the reflectance of the PE layer on a Ag layer via ray-tracing method ^2^, as shown in Fig. S11. Here, we ignore the internal interference effect between the PE surface and the PE-Ag boundary due to the existing of microchannels inside the multilayered film. The optical constants (refractive index *n* and extinction coefficient *κ*) of PE was obtained from Ref. 3 ^3^. The thickness of the PE film is assumed to be *d*, and the reflection of the PE-Ag boundary is assumed to be 1. The surface reflectance *ρ_λ_* and internal transmittance *τ_λ_* can be calculated as

$\rho_{\lambda}=\frac{{(n-1)}^{2}+k^{2}}{{(n+1)}^{2}+k^{2}}$ (6)

$\tau_{\lambda}=exp(-\frac{4\pi kd}{\lambda}$) (7)

Based on the ray tracing, the spectral reflectance *R*_λ_ can be obtained by

$$R_{\lambda}=\rho_{\lambda}+{\rho_{\lambda}\left( 1-\rho_{\lambda} \right)}^{2}{\tau_{\lambda}}^{2}+{{\rho_{\lambda}}^{3}\left( 1-\rho_{\lambda} \right)}^{2}{\tau_{\lambda}}^{4}+{{\rho_{\lambda}}^{5}\left( 1-\rho_{\lambda} \right)}^{2}{\tau_{\lambda}}^{6}+\ldots$$

$=\rho_{\lambda}{[1+\frac{{(1-\rho_{\lambda})}^{2}+{\tau_{\lambda}}^{2}}{1-{\rho_{\lambda}}^{2}{\tau_{\lambda}}^{2}}]}$ (8)

As the transmittance of Ag layer is zero, the spectral absorptance *A*_λ_ can be calculated as

$A_{\lambda}=1-R_{\lambda}$ (9)

According to Kirchhoff’s law, the spectral emittance *ε*_λ_ equals to its absorptance in thermodynamic equilibrium:

$\varepsilon_{\lambda}=A_{\lambda}$ (10)

Finally, the average hemispherical emissivity$\epsilon$ in the atmospheric transmittance window (7.5-14 μm) can be obtained by

$\epsilon= \frac{\int_{7.5 \mu m}^{14 \mu m} {I_{B, \lambda}\varepsilon}_{\lambda}d\lambda}{\int_{7.5 \mu m}^{14 \mu m} I_{B,\lambda}d\lambda}$ (11)

𝐼*_B_*_,𝜆_ is the spectral radiation intensity of blackbody at room temperature.

**3. Mimicking the reflectance spectra of plant leaves**

Natural leaves, as a common background for camouflage targets, have very typical spectral characteristics, including a reflection peak around 550 nm, a high near-infrared reflection plateau between 800-1400 nm, and two water absorption valleys around 1450 nm and 1940 nm ^4^. These spectral features mainly result from the complicate interactions between incident light and the elaborate porous architectures and functional components such as pigments and water ^5^. Thus, it is still difficult to mimic the broadband reflective spectra of leaves from visible to the end of near infrared region.

Here, we designed the microfluidic film to exhibit similar spectra as natural plant leaves as shown in Fig. S15a. The top microchannel layer contains colored aqueous fluids for color control. The bottom microchannel layer contains grey aqueous fluids for reflective intensity modulation. The paper at the bottom provides a static diffusive reflection via porous structure. The width and depth (~640 μm × 100 μm) of the microchannels in each layer can be found in Fig. S15b.

Supplementary Figures


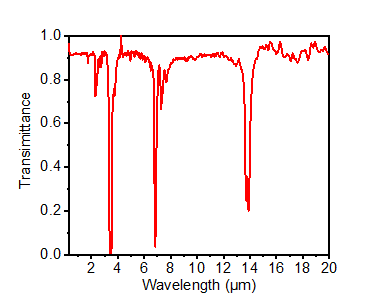


**Fig. S1** Spectral transmittance of a 30-μm-thick low-density polyethylene film in the wavelength range from 0.3 μm to 20 μm.


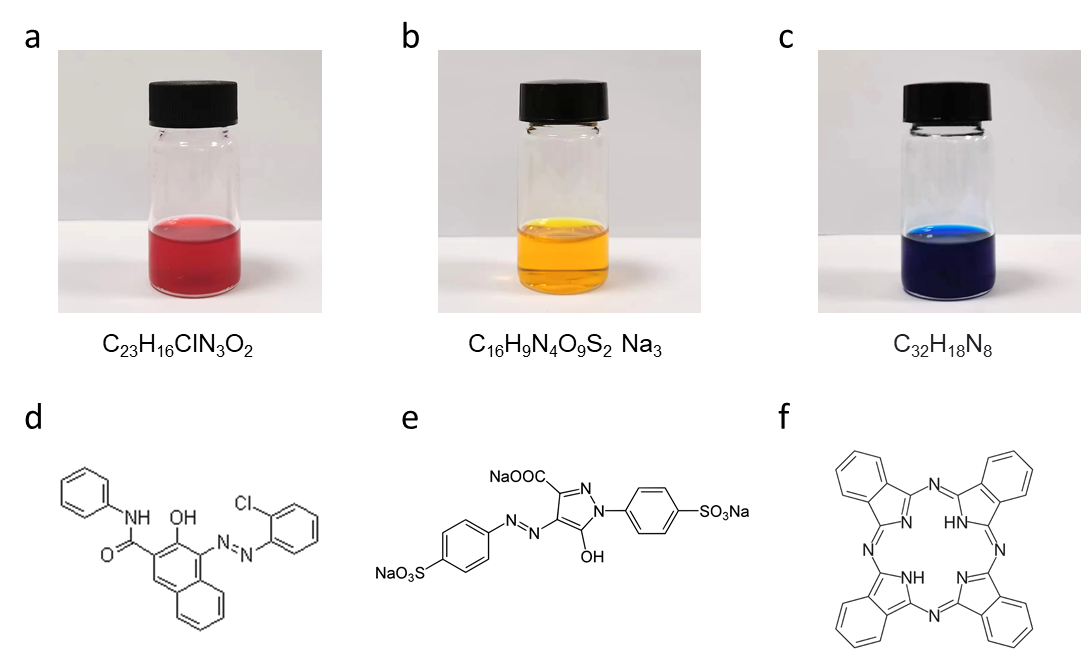


**Fig. S2** Three primary fluids. **a**, **b**, **c**, Photographs of red (**a**), yellow (**b**) and blue (**c**) fluids. **d**, **e**, **f**, Molecular structures of the C_23_H_16_ClN_3_O_2_ (**d**), C_16_H_9_N_4_O_9_S_2_Na_3_ (**e**) and C_32_H_18_N_8_ (**f**) dyes.


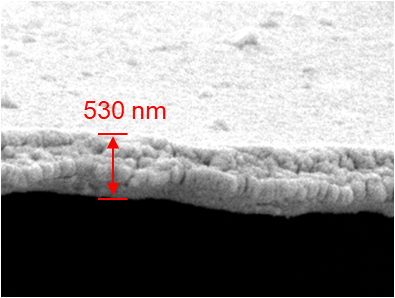


**Fig. S3** Cross-sectional SEM image of the Ag layer with the thickness of 530 nm.


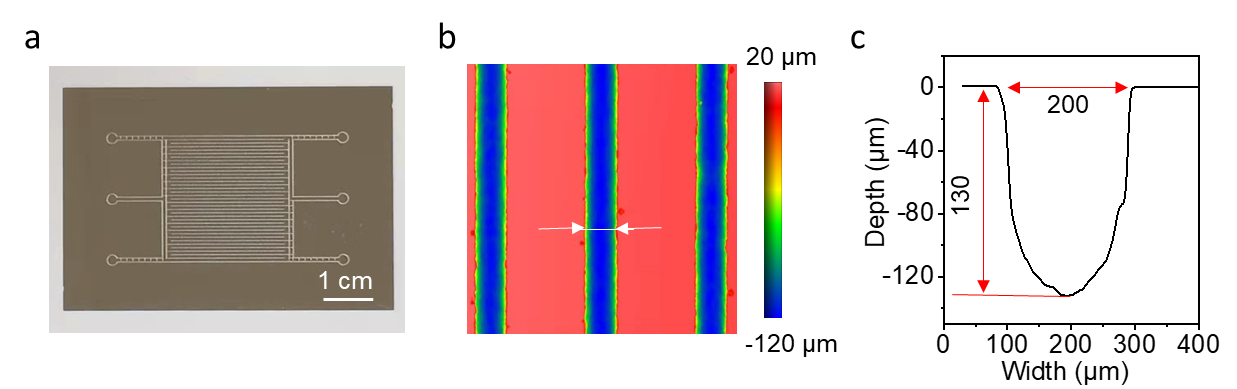


**Fig. S4** Characterization of the mold. **a**, A photograph of the microchannel mold fabricated on a stainless-steel sheet. **b**, Surface morphologies of microchannel. **c**, The width and depth of the microchannel measured from the position marked by the two arrows in **b**.


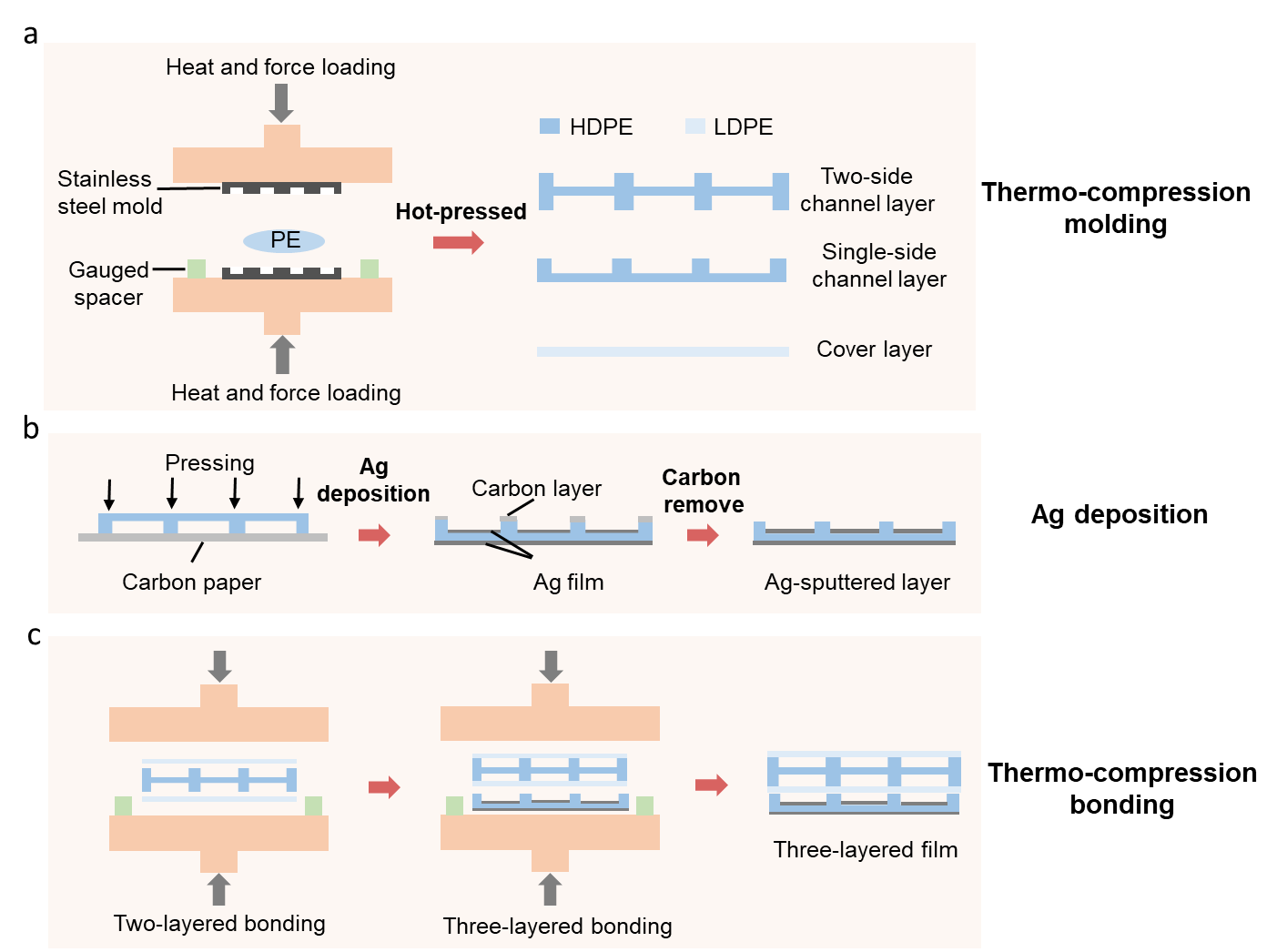


**Fig. S5** Schematic of fabrication processes of the microfluidic film with three-layered microchannels.


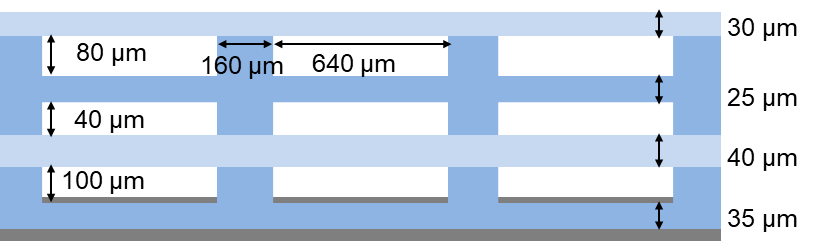


**Fig. S6** Structure and sizes of the three-layered microchannels estimated from the SEM image in Fig. 2c.


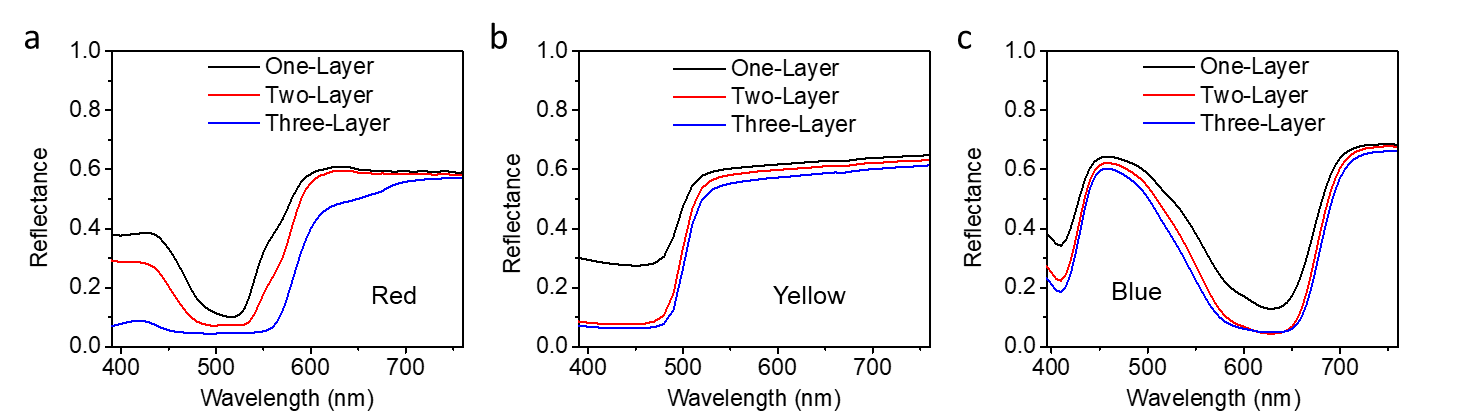


**Fig. S7** Spectral reflectance of the red (**a**), yellow (**b**) and blue (**c**) microfluidic film with different thickness.


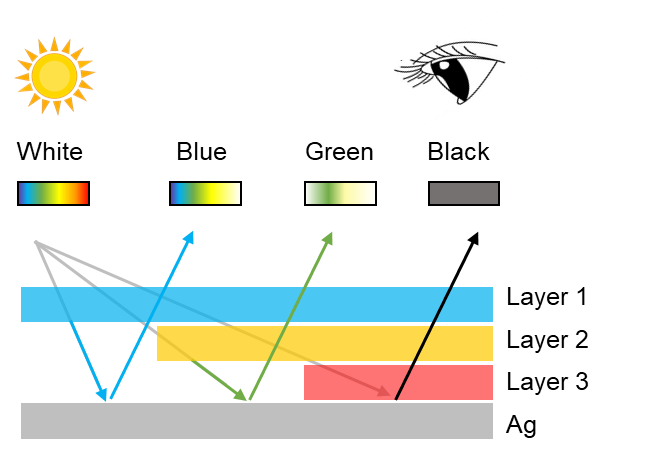


**Fig. S8** Principle of subtractive color mixing for the three-layered microfluidic film.


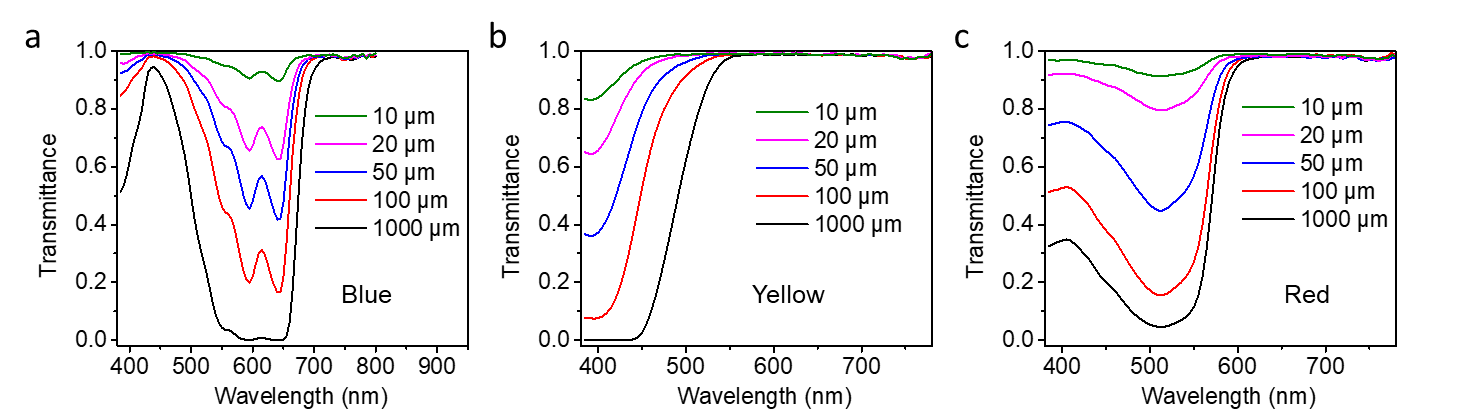


**Fig. S9** Transmittance of the blue (**a**), yellow (**b**) and red (**c**) fluids with different thicknesses.


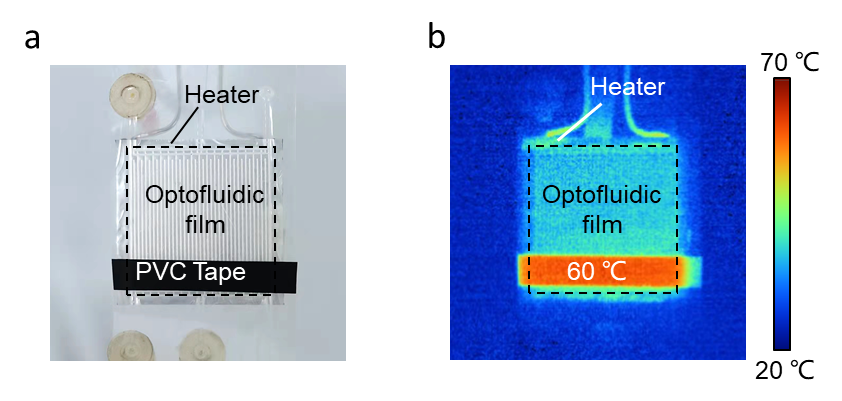


**Fig. S10** Experimental setup for the thermal emissivity measurement. The black PVC tape with an emissivity of 0.91 was used for reference.


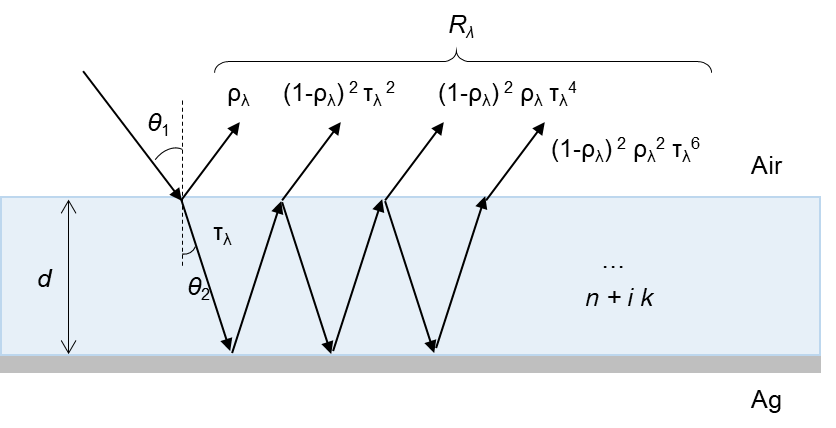


**Fig. S11** Schematic of the reflectance in a single layer with a back reflective layer.


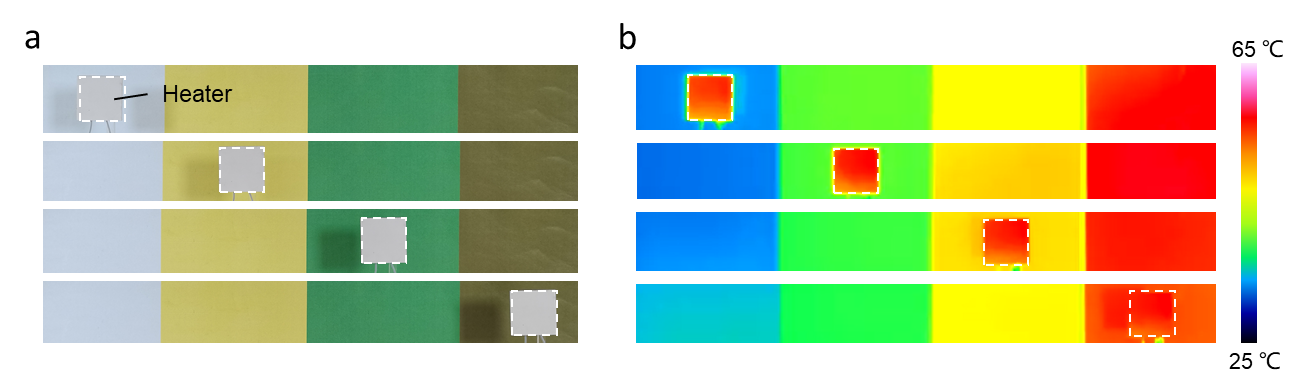


**Fig. S12** Visible (**a**) and infrared (**b**) views of the ceramic heater without microfluidic film moving across the background with varying colors and temperatures.


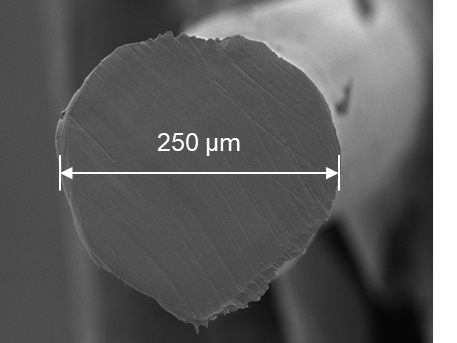


**Fig. S13** Cross-sectional SEM image of the solid PE fiber.


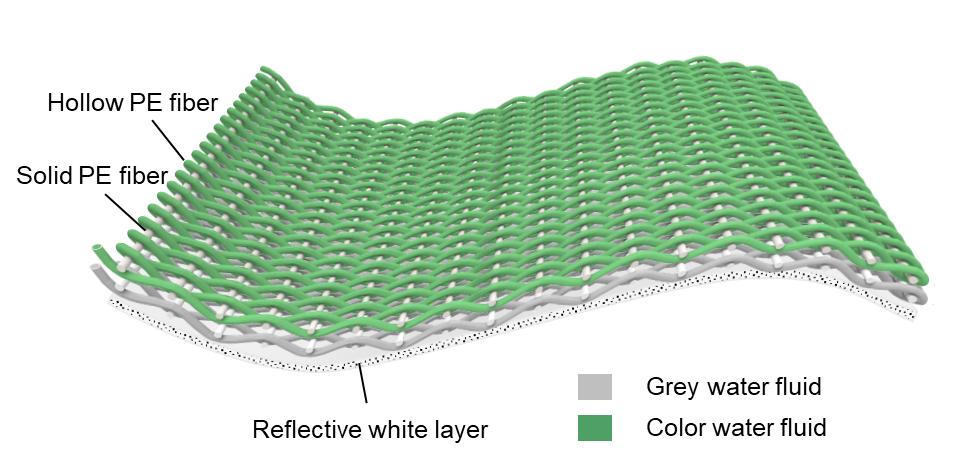


**Fig. S14** Schematic showing the structure of the optofluidic textile constructed by hollow PE fibers and solid PE fibers.


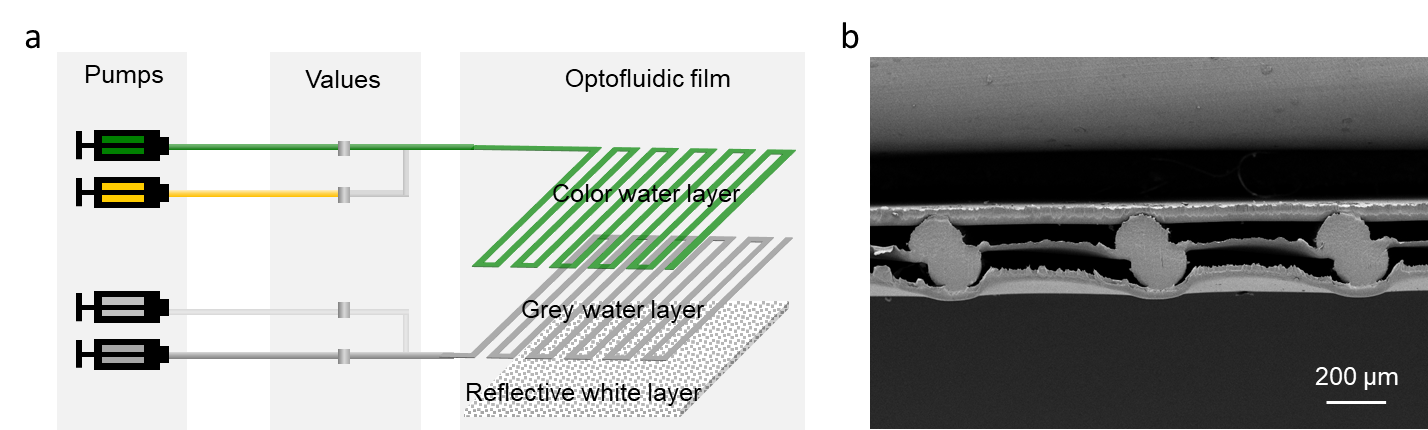


**Fig. S15** **a**, Schematic of operation of the two-layered microfluidic film for mimicking the natural leaves. **b**, Cross-sectional SEM image of the two-layered microfluidic film.


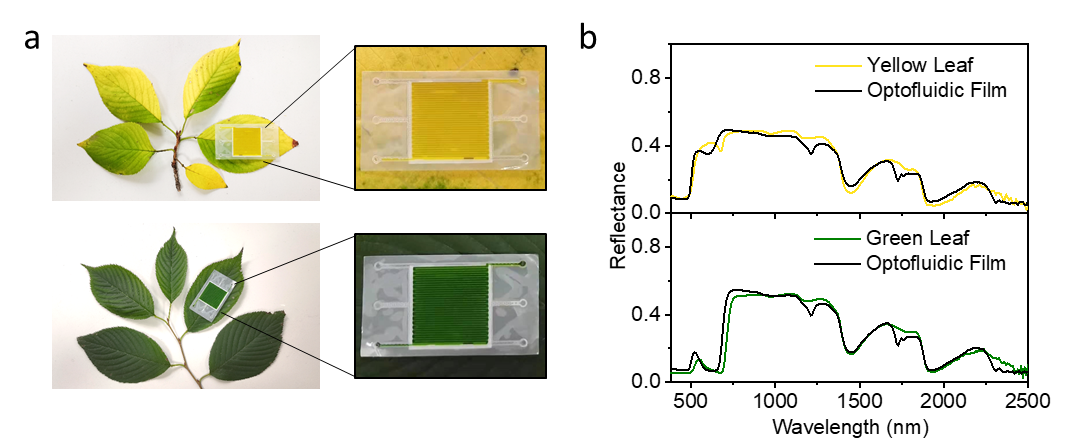


**Fig. S16** **a**, Photographs show that the two-layered microfluidic film can change its visual appearance to match leaves with yellow and green colors. **b**, Reflectance spectra of the microfluidic film and leaves in the range of 400-2500 nm.


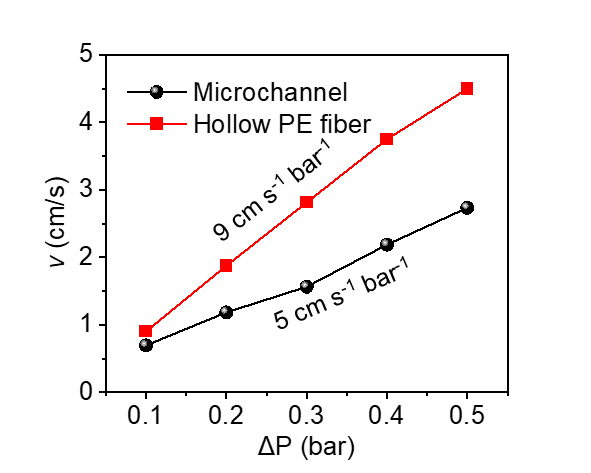


**Fig. S17** Flow rates of the microchannel and hollow PE fiber under different applied pressure. The microchannel has the width of ~640 μm and height of ~100 μm. The hollow PE fiber has an inner diameter of 300 μm and wall thickness of 25 μm.


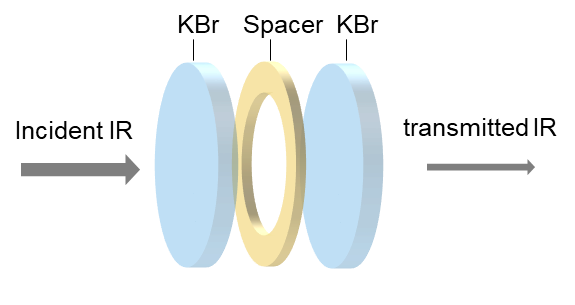


**Fig. S18** Schematic showing the experimental setup for measuring IR transmittance of the fluids. Thickness of the fluid can be set by the spacer.

Supplementary table

**Table. S1** Arrays corresponding to the fluid states in the film

|  | **ε** | **White** | **Red** | **Yellow** | **Blue** | **Orange** | **Green** | **Violet** | **Black** |
| --- | --- | --- | --- | --- | --- | --- | --- | --- | --- |
| **Empty** | 0 0 0 | 0 0 0 |  |  |  |  |  |  |  |
| **Single**  **layer** | a 0 0 |  | 1 0 0 | 2 0 0 | 3 0 0 |  |  |  |  |
|  | 0 b 0 |  | 0 1 0 | 0 2 0 | 0 3 0 |  |  |  |  |
|  | 0 0 c |  | 0 0 1 | 0 0 2 | 0 0 3 |  |  |  |  |
| **Double**  **layers** | a b 0 |  | 1 1 0 | 2 2 0 | 3 3 0 | 1 2 0  2 1 0 | 1 3 0  3 1 0 | 2 3 0  3 2 0 |  |
|  | a 0 c |  | 1 0 1 | 2 0 2 | 3 0 3 | 1 0 2  2 0 1 | 1 0 3  3 0 1 | 2 0 3  3 0 2 |  |
|  | 0 b c |  | 0 1 1 | 0 2 2 | 0 3 3 | 0 2 3  0 3 2 | 0 1 3  0 3 1 | 0 2 3  0 3 2 |  |
| **Three**  **layers** | a b c |  | 1 1 1 | 2 2 2 | 3 3 3 | 1 1 2  1 2 1  1 2 2  2 1 1  2 1 2  2 2 1 | 1 1 3  1 3 1  1 3 3  3 1 1  3 1 3  3 3 1 | 2 2 3  2 3 2  2 3 3  3 2 2  3 2 3  3 3 2 | 1 2 3  1 3 2  2 1 3  2 3 1  3 1 2  3 2 1 |

Supplementary videos

Video 1 Color switching from yellow to blue then to red in the microfluidic film.

**Video 2 and 3** Camouflage demonstration of the microfluidic film in visible and mid-infrared region.

Supplementary References

1. Fairman, H. S., Brill, M. H. & Hemmendinger, H. How the CIE 1931 color-matching functions were derived from Wright-Guild data. *Color Research & Application* **22**, 11-23 (1997).

2. Lee, H. & Zhang, Z. Applicability of phase ray-tracing method for light scattering from rough surfaces. *Journal Of Thermophysics and Heat Transfer* **21**, 330-336 (2007).

3. Palik, E. D. *Handbook of optical constants of solids*. Academic press (1998).

4. Woolley, J. T. Reflectance and transmittance of light by leaves. *Plant Physiology* **47**, 656-662 (1971).

5. Knipling, E. B. Physical and physiological basis for the reflectance of visible and near-infrared radiation from vegetation. *Remote Sensing Of Environment* **1**, 155-159 (1970).
